# Hatching phenology is lagging behind an advancing snowmelt pattern in a high-alpine bird

**DOI:** 10.1038/s41598-021-01497-8

**Published:** 2021-11-12

**Authors:** Christian Schano, Carole Niffenegger, Tobias Jonas, Fränzi Korner-Nievergelt

**Affiliations:** 1 Swiss Ornithological Institute, 6204 Sempach, Switzerland; 2 Department of Evolutionary Biology and Environmental Studies, University of Zurich, 8057 Zurich, Switzerland; 3 Snow Hydrology, WSL Institute for Snow and Avalanche Research SLF, 7260 Davos Dorf, Switzerland

**Keywords:** Phenology, Ecology

## Abstract

To track peaks in resource abundance, temperate-zone animals use predictive environmental cues to rear their offspring when conditions are most favourable. However, climate change threatens the reliability of such cues when an animal and its resource respond differently to a changing environment. This is especially problematic in alpine environments, where climate warming exceeds the Holarctic trend and may thus lead to rapid asynchrony between peaks in resource abundance and periods of increased resource requirements such as reproductive period of high-alpine specialists. We therefore investigated interannual variation and long-term trends in the breeding phenology of a high-alpine specialist, the white-winged snowfinch, *Montifringilla nivalis*, using a 20-year dataset from Switzerland. We found that two thirds of broods hatched during snowmelt. Hatching dates positively correlated with April and May precipitation, but changes in mean hatching dates did not coincide with earlier snowmelt in recent years. Our results offer a potential explanation for recently observed population declines already recognisable at lower elevations. We discuss non-adaptive phenotypic plasticity as a potential cause for the asynchrony between changes in snowmelt and hatching dates of snowfinches, but the underlying causes are subject to further research.

## Introduction

To maximise reproductive success, animals need to rear their offspring when conditions are most favourable^1^. Many species utilize seasonally fluctuating food sources and thus need to time their reproduction to often relatively short periods of optimal conditions to avoid severe fitness consequences^2^. In birds, timing of reproduction is further complicated by the fact that non-tropical species regress their gonads outside of the reproductive season^3^. They thus need to induce gonadal growth ahead of optimal conditions to reproduce and lay eggs^4,5^.

While photoperiod often serves as a predominant, proximate cue to induce gonadal growth and the onset of breeding^3–6^, the precise timing of egg-laying and hatching will depend on local environmental conditions^7^. Birds therefore use further environmental cues to better predict the nesting season in advance^8–10^. However, the reliability of such cues may be impaired by climate change i.e., when cue and food source respond differently to the changing environment ^11,12^. Climate change may therefore especially affect ecological specialists with narrow thermal niches and ephemeral food sources^13,14^.

As such, arctic-alpine habitats and their usually cold-adapted residents experience strong seasonality, predominantly determined by low temperatures^15^ which then cause prolonged periods of snow cover^16^ and short vegetation periods^17^. Arctic-alpine habitats currently experience broad landscape change^18^, disproportionate warming^19–21^, shifts in seasonal, and elevational precipitation^22,23^, an increase of extreme weather events^24^ and changing snow cover durations^25^. As such, the strong seasonality of temperate high elevation habitats and differences in response to climate change between trophic levels thus constantly challenge arctic-alpine bird species^26^. Invertebrate availability quickly increases after snowmelt initiation and arctic and alpine species benefit from timing their broods so that chicks grow during peak invertebrate availability^27–29^. Although the relationship between nesting and snowmelt is evident for some arctic waders^30^ and passerines^31^, comparatively few studies focus on the role of snowmelt on the breeding phenology in alpine bird species^32,33^.

We therefore studied the role of snowmelt and other possible environmental cues on the breeding phenology of a high-alpine specialist, the white-winged snowfinch, *Montifringilla nivalis* (henceforth snowfinch). To do so, we analysed a data set on snowfinch hatching spanning 20 years to investigate (1) the proportion of broods hatching during snowmelt, (2) long-term changes of hatching dates and (3between-year variability of hatching dates as a function of environmental predictors. Snowfinches predominantly rear their offspring on larval tipulids whose availability strongly correlate with snow cover^34^. Snowfinches should thus benefit from breeding once the snow starts to melt, and we would expect a large proportion of broods to hatch during snowmelt and the average hatching date to advance in unison with a potential advance in snowmelt timing. We thus expected that hatching dates (a) advance with higher temperatures, even after correcting for snow condition and elevation because warm temperatures foster gonadal development, (b) delay with increased precipitation, because it compromises pre-breeding conditions, (c) advance with earlier snowmelt initiation independent of temperature and elevation, because the ocurrence of snow-free patches may be used as a cue to anticipate the timing of the peak food availability during snowmelt, and (d) delay after harsh winters, because adults may be in low body condition after more demanding winters.

## Results

### Breeding phenology in relation to snow cover fraction

**Figure 1:**
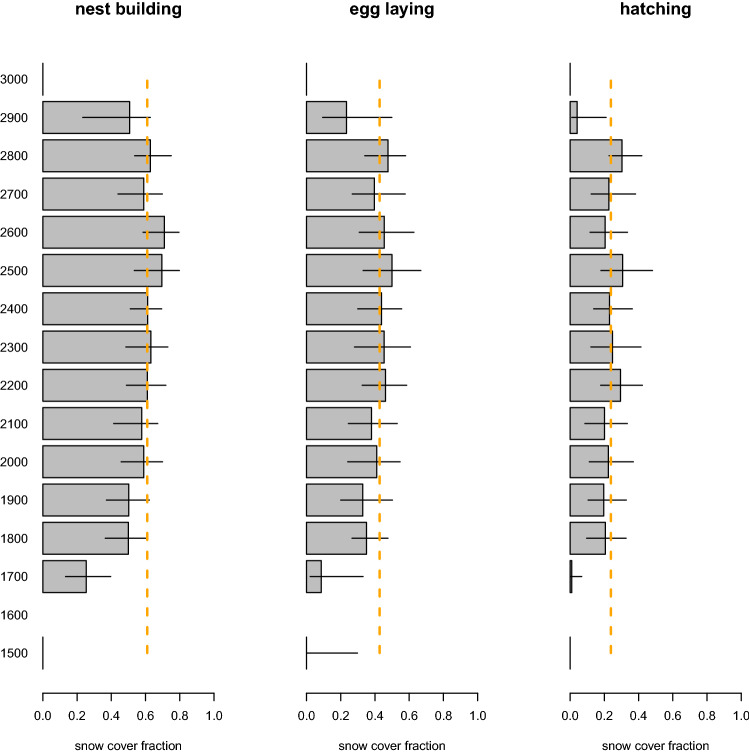
Mean snow cover fraction per elevation during nest building, egg laying and hatching. Error bars range from average earliest to average latest possible dates per event, orange lines (dotted) indicate mean snow cover fraction per event.

On avagerage, snowfinches built nests when snow cover fraction was 61% (range: 48–71%), layed eggs at around 43% (range: 28–57%) and hatched at 24% (range: 13–38%) based on 1135 broods and an annual mean of 57 (SD ± 42) broods (Fig. 1). Only a minority of 7.11% (range: 0.53–17.78%) of snowfinches built nests before meltstart, and 1.07% (range: 0.36–3.91%) layed eggs before meltstart. Nesting after the snow had melted completely was also rare: 9.6% (range: 4.09–29.78%) of nests and 19.64% (range: 11.02–32.27%) of egg laying were initiated after meltend. Thus, most snowfinches built nests, layed eggs and eggs hatched during the snowmelt, which, on average, lasted for 70 days (SD ± 16 days) in 1 × 1 km grid cells with snowfinch observations. Nearly two thirds, 64.22% (range: 46.87–76.48%) also hatched during the snowmelt. The overall mean hatching date was June 24th (SD ± 17 days), but annual average hatching dates ranged from June 16th (SD ± 15 days) in 2017 to July 4th (SD ± 11 days) in 2013. At the elevational centre of their distribution, snowfinches bred at higher snow cover compared to broods at the edges of their elevational distribution.

### Long-term changes in snowmelt and hatching phenology

**Figure 2:**
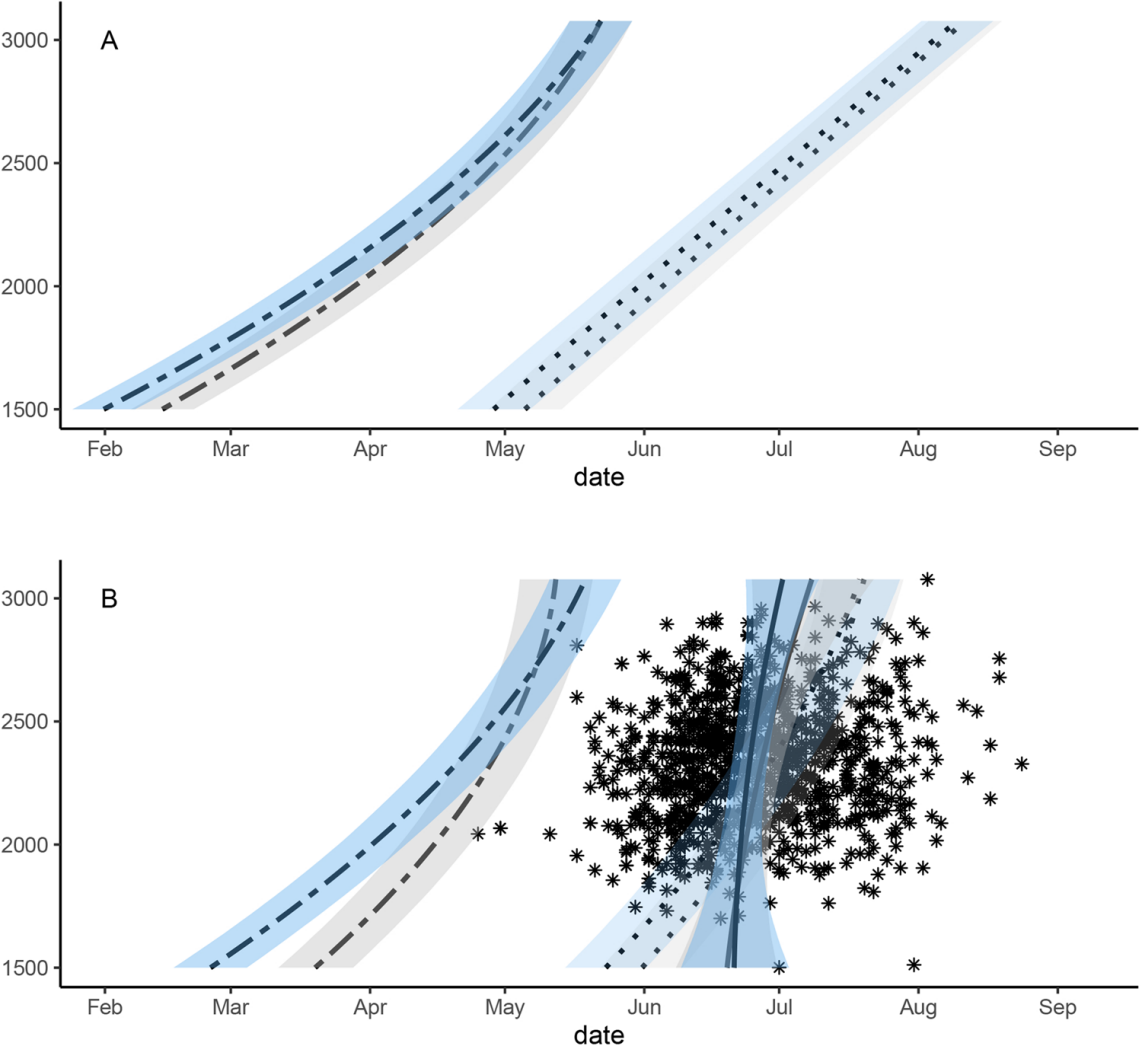
Mean dates for snowmelt initiation (twodash), snowmelt completion (dotted) and snowfinch hatching dates (solid) along the elevational gradient for 1999 (grey) and 2018 (blue), based on all snowfinch data with snowmelt estimated based on a complete dataset of Swiss kilometre grid cells between 1500 and 3100 m **(A)** and those with snowfinch observations **(B)**. Shaded areas represent 95% Bayesian compatibility intervals.

Although mean meltstart advanced from April 11th (CI: April 4th–18th) in 1999 to April 5th (CI: March 29th–April 11th) in 2018, snowfinches did not advance hatching date at elevations below 2000 m. However, average hatching date was earlier at high elevations even though snowmelt delayed. Further, snowfinches bred in 1 × 1 km grid cells with significantly later snowmelt compared to grid cells without snowfinch observations (Fig. 2A,B). In 1999, the estimated hatching date was 65 (range: 50–80) days after meltstart but in 2018, it had prolonged to 71 (range: 60–85) days after meltstart. Relative to meltstart, snowfinches generally bred later at lower elevations compared to higher elevations. This difference became more pronounced towards the end of the study period. Changes in average hatching dates were largest at high elevations, whereas they were smallest below 2200 m asl with mean changes of less than a day per decade.

### Spatiotemporal pattern in hatching date

**Figure 3:**
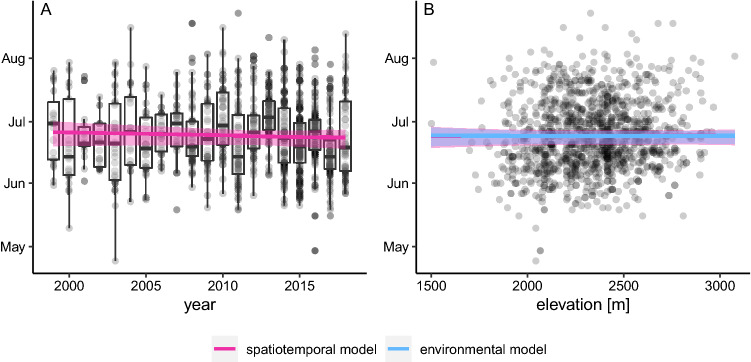
Hatching date of snowfinches in relation to year **(A)** and elevation **(B)** between 1999 and 2018. Lines represent regression lines of a model including spatiotemporal predictors (pink) and from a model also including environmental predictors (blue). Polygons show Bayesian 95% compatibility intervals accordingly. In **(A)**, we only show the regression line for the spatiotemporal model because the regression lines of the two models were essentially identical.

Given observers would be active at the same dates, mean hatching dates at an elevation of 2450 m asl varied between biogeographic regions, being earliest in the Western Alps (June 19th, CI June 17th–June 22nd) and latest in the Southern Alps (June 26th, CI June 23rd–June 29th) (Table 1). The conditional mean hatching dates in the Eastern Alps (June 24th, CI June 22nd–June 26th) and the Northern Alps (June 25th, CI June 23rd–June 27th) were more similar to the Southern Alps. We found a clear positive correlation between mean hatching date and observer date (2.06, CI: 1.86–3.21 days). Average hatching dates advanced over the years (Fig. 3A), more strongly so at higher elevations, although CIs for the coefficents of elevation and year were broad (Fig. 3B).

**Table 1:** Standardised and unstandardised parameters for the spatiotemporal and environmental model including Bayesian compatibility intervals and standard deviations.

Parameters	Spatiotemporal model		Environmental model		Raw data
	β (SE) standardised	β (SE) unstandardised	β (SE) standardised	β (SE) unstandardised	SD
Region [EA]	–	175.29 (1.06)	–	175.50 (1.03)	15.73 days
Region [NA]	–	176.36 (1.13)	–	175.84 (1.06)	18.48 days
Region [SA]	–	177.57 (1.53)	–	176.90 (1.49)	16.13 days
Region [WA]	–	170.93 (1.14)	–	171.67 (1.07)	17.87 days
Elevation	0.73 (3.74)	0.3 (1.54) 100 m^−1^	− 0.6 (3.86)	−0.25 (1.59) 100 m^−1^	243.22 m
Elevation^2^	0.32 (3.71)	0.13 (1.53) 100 m^−1^	0.92 (3.76)	0.38 (1.55) 100 m^−1^	–
Mean observer day	8.24 (0.46)	0.49 (0.03) day^−1^	8.17 (0.46)	0.48 (0.03) day^−1^	16.97 days
Year	− 0.66 (0.73)	−1.33 (1.48) decade^−1^	−0.68 (0.66)	−1.38 (1.33) decade^−1^	4.94 years
Elevation:year	−0.06 (0.44)	−0.05 (0.37) 100 m^−1^ decade^−1^	−0.12 (0.45)	−0.1 (0.37) 100 m^−1^ decade^−1^	
Winter intensity	–	–	−0.05 (0.76)	−0.05 (0.76)	1.18 (no unit)
Precipitation	–	–	1.14 (0.58)	0.9 (0.46) 100 mm^−1^	2.08 mm/day
Snowmelt initiation	–	–	0.6 (0.53)	0.18 (0.16) week^−1^	22.83 days
Temperature	–	–	−1.05 (1.03)	−0.5 (0.49) °C^−1^	2.11° C
Among-year standard deviation		2.7 (CI: 1.5–4.4)		1.9 (CI: 0.3–3.7)	

Unstandardised parameters show change in hatching date per unit of the predictor. Winter intensity has no unit because it is measured as a principal component of temperature and precipitation.

### Hatching date in relation to environmental variables

**Figure 4:**
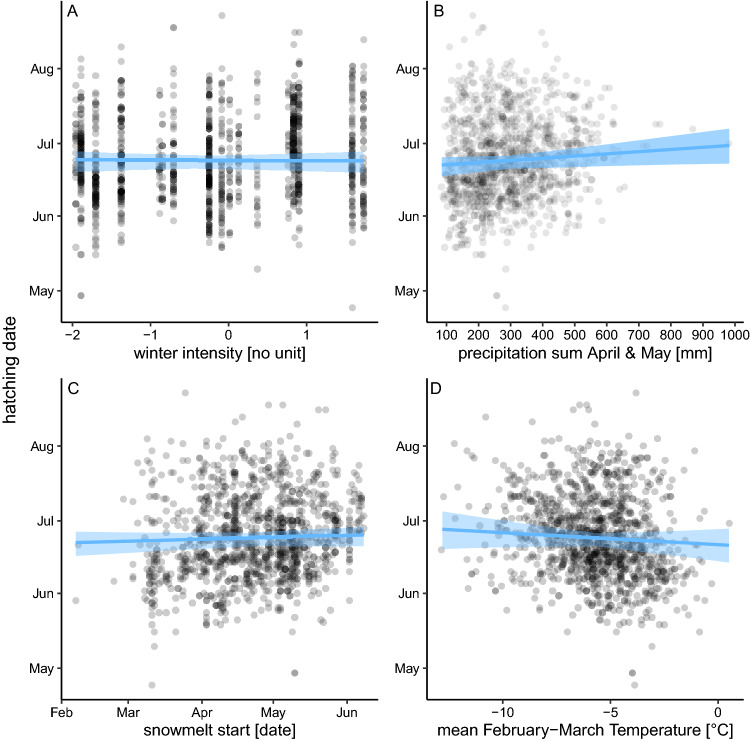
Hatching date of snowfinches in relation to partial effect sizes of winter intensity **(A)**, precipitation **(B)**, snowmelt initiation **(C)** and temperature **(D)** as estimated from a model including both spatiotemporal and environmental predictors. Blue polygons show Bayesian 95% compatibility intervals.

Precipitation showed a clear positive relationship with snowfinch hatching dates (1.14; CI: 0.001, 2.28), thus suggesting a one-day delay in hatching date with 111 mm of additional precipitation between April and May (CI: 38.47, 672.64 mm) (Fig. 4B). Partial correlations of all other environmental variables showed broad compatibility intervals indicating that these relationship are difficult to measure (Fig. 4A,C,D).

### Observer bias

To assess observer phenology on the observed brood phenology, we investigated observer behaviour along the elevational gradient and snowmelt patterns. We compared spatiotemporal observer distribution with the distribution of hatching dates using bivariate normal kernel densities^35^ (Supplementary 1). Less than half the observers, 44.58%, were present during snowmelt. Observers covered the entire breeding season and exceeded the 95% mass distribution of snowfinches both before and after the peak of the breeding season. Relative to snowmelt, 95% of observers entered data between 20 days before and 180 days after the snowmelt and thus covered snowfinch broods well, as these mostly hatched between 2 and 127 days after snowmelt.

## Discussion

### Breeding phenology in relation to snow cover fraction

Our results support the many former studies that showed how strongly the nesting timing of snowfinches are connected to snowmelt. Snow cover is an important modulator in ecological systems across mountain ranges and often affects breeding performance in alpine specialists^32,36,37^. Snowfinches may feed on arthropod fallout on snow patches but particularly forage for larval arthropods, only accessible once the snow melts^34,38,39^. Snow cover may thus proximately predetermine laying and hatching dates, limited by the availability of nesting material or protein-rich prey for egg development and feeding offspring^32^. Our results suggest only a small fraction of laying events (range: 0.36–3.91%) to occur before meltstart. Further, snowfinches lay eggs at about 43% and hatch at about 23% snow cover. They may thus particularly exploit early peaks in food abundance, specifically tipulid larvae, which emerge beneath melting snow and overproportionally contribute to early spring arthropod biomass^29,34^.

On average, two thirds of snowfinch broods hatched during the snowmelt period and the mean hatching date was around June 25th in 2011 for the Northern Alps, translating to an approximate laying date around June 12th at 2450 m asl. This is considerably later than reported by Heiniger in 1991^40^ for the Bernese Alps (Northern Alps) where he suggested snowfinches to lay eggs independent of temperature and the snow situation from the second half of May. However, Heiniger^40^ visited previously known, small-scale breeding sites and likely only included first broods. In contrast to Heiniger’s method, we inferred hatching dates from the behaviour of adults. Therefore, broods that did not hatch are likely underrepresented in our data. Additionally, our method cannot distinguish between first and second broods, possibly resulting in later average hatching dates than Heiniger^40^ suggested.

### Long-term changes in snowmelt and hatching phenology

Since the breeding and foraging behaviour of birds are ultimately determined by favourable physiological conditions and food availability^41^, we would expect hatching dates to change over time in proportion to environmental conditions that affect food abundance. However, we found average hatching date to be stable at low elevations, even though meltstart advanced considerably. In contrast, hatching slightly advanced at higher elevations although meltstart delayed over the course of two decades. Previous studies have shown that phenological shifts in larval arthropods correspond to long-term snowmelt patterns^34,42^. Therefore, non-parallel long-term changes in snowmelt and hatching date would suggest that there are drivers of change in mean hatching dates which are not synchronised with the timing of peaks in food abundance. We can think of three, not mutually exclusive explanations for heterogeneous shifts of snowfinch hatching dates along an elevational gradient.

Firstly, an advance in average hatching date could be caused by a decrease in the proportion of multiple broods at the respective elevations. Snowfinches are facultatively double-brooded, but the proportion of double brooded pairs in snowfinches may be highly variable in space and time. Snowfinches may skip breeding in years with late snowmelt, whereas in other years they can breed twice^40^. In the French Pyrenees, 50% of the breeding pairs are double brooded^43^ and in the Abruzzi mountains, all pairs breed twice^44^. The proportion of second broods in our data is unknown, but it could have decreased over time either due to a reduced number of pairs producing second broods, or by a larger proportion of undetected abandoned or failed second broods. A decreasing proportion of second broods could probably also explain largely constant hatching dates at lower elevations, as a reduction of second broods may be associated with a delay of the first brood towards a peak in food availability when snowfinches were historically adapted to start the first brood well before the peak food availability to favor second broods^45^. However, average hatching date at low elevations was connected with strongly decreasing proportions of broods hatching during the snowmelt and thus, the peak food availability. Secondly, a constant average hatching date at low elevation could result from simultaneously advancing the first brood and increasing the proportion of second broods. However, because the proportion of broods hatching during snowmelt strongly decreased (see results) and we have no indication that the duration of the breeding season increased over the years (Supplementary 2), we can exclude that increasing proportions of second broods blur an advance in the observed average hatching date at elevations below 2000 m. At higher elevations, a decreasing proportion of second broods likely explains the slight advance in average hatching date because it is unlikely that snowfinches start breeding earlier relative to snowmelt. Lastly, average hatching dates could simply not have changed over time, whereas snowmelt advanced at elevations below 2000 m across the study period. The increasing temporal lag between snowmelt and hatching date may have resulted in deteriorating breeding conditions, which may explain the observed population decline at low elevations^46^.

### Environmental parameters influencing breeding phenology

We found considerable among-year variation in mean hatching dates, indicating that snowfinches use flexible cues to respond to variability in environmental conditions. Our results suggest spring precipitation (April–May) to be an important environmental factor that potentially serves as a modulator for breeding phenology^47,48^. Extreme weather events like heavy snowfall or extreme temperature decreases at the beginning of the breeding season can affect the breeding performance of arctic and alpine species drastically^49^.

April and May precipitation in snowfinch breeding habitat is largely comprised of snowfall and therefore strongly determines the conditions experienced during the courtship and brood initiation phase. Snowfinches may suffer from spontaneous snow onset by losing access to food resources or being forced to travel larger distances to provide offspring with food^49^. Spring precipitation may thus explain the rather large among-year variation in mean hatching dates, indicating that snowfinches use spring precipitation as an environmental cue to predict the onset of breeding.

For the other variables, snowmelt, temperature and winter intensity, uncertainty in the partial correlation coefficients were high. Our results regarding these variables therefore do not indicate strong partial correlations with the brood initiation of snowfinches but may still bear consequences for snowfinches and thus need further research.

Winter intensity could affect snowfinch breeding behaviour in two ways. On the one hand, harsh winters may delay breeding initiation or force snowfinches to skip breeding entirely^40,50^, therefore prioritising individual survival^51^. On the other hand, snow-rich winters are associated with good thermal insolation, little soil frost and late meltend, resulting in warm soil temperatures after meltend. This may have a positive effect on larval tipulid abundance overwintering under the snow and could thus assure food supply for snowfinches to find once the snow melts. Further, spring temperature is a known modulator for critical phases during the breeding season, specifically so during gonadal recruitment and egg laying but may also hold negative effects on populations thereafter^8^.

### Observer effect

Often, citizen science data is the only source of information available for specific biological measures. Hence, they are invaluable for the description of long-term trends and large-scale trends in populations^52^ or phenology^53^, especially in difficult terrain where monitoring data is still scarce^54^. However, citizen science data is often characterised by heterogeneous observer activity and, therefore, patterns in observer activity need to be separated from patterns in the biological measure of interest before conclusions can be drawn. We assessed observer influence on two different scales. First, we compared observer distribution against hatching date distribution in relation to elevation and date. We argue that, in comparison to hatching dates, observers were wider and more homogeneously distributed along these two axes, suggesting an underlying biological rather than an observational cause. Secondly, we accounted for observer bias by including mean observer day as a predictor in the models analysing hatching dates. Our results indicate a strong relationship between observer and hatching day, hence emphasising the importance of accounting for observer bias, especially in difficult alpine terrain.

### Potential Implications of climate change on high-alpine specialists

We analysed a long-term dataset of a high-alpine bird specialist and found among-year variance in hatching dates, correlated with April and May precipitation. Among-year variability in hatching dates suggest breeding snowfinches to be able to adjust the timing of their broods to current environmental conditions, but a long-term trend in average hatching date did not coincide with a long-term trend in snowmelt phenology. Growing scientific evidence suggests arctic^55^ and alpine^56^ vertebrates to have comparatively low genetic diversity and adaptive phenotypic plasticity may only enable high-alpine specialists to cope with meteorological variation below certain thresholds^50,57^. Evidence for microevolutionary changes in breeding phenology^57^, especially in alpine birds, is still scarce and we thus highlight the importance of further research on the evolutionary adaptation in alpine specialists. Our results may suggest a mismatch between the broods and the time of assumed peak food availabiltiy at low elevations in the Alpine population of snowfinches. Potential carry-over effects of such a mismatch may affect later stages of life, including breeding success^50^ and parental survival^41^. This may explain why snowfinch populations exceedingly decline at elevations below 2000 m asl^46^ and is in line with findings on Pleistocene fossil records indicating that species were more prone to shift their distribution ranges rather than tracking climate change despite much more drastic warming^26^. Snow cover duration in the Swiss Alps has, below 2500 m asl and regardless of region or elevation, declined by an average of 8.9 days per decade since the 1970s, mainly due to earlier snowmelt^58^. Under current climate change scenarios, hatching dates may thus further dissociate from snowmelt initiation at lower elevations and potentially have severe fitness effects on snowfinches. Although the specific mechanisms are still largely unclear, warming temperatures might threaten high-alpine bird species in several ways^59^. As such, snowfinches are specifically sensible to warming temperatures and physiological consequences like hyperthermia have previously been hypothesised as a potential cause of lower female survival^60^. Higher temperatures may alter plant composition, growth speed and drying of alpine meadows^61^ and thus decrease food availability for snowfinches, increasing energy expenditure of feeding adults. Additionally, current climate models suggest an increase in extreme weather events^48,49^, possibly exceeding the physiological thresholds of alpine specialists at some point. Our results suggest that precipitation at an early stage of the breeding season may affect breeding phenology. However, little is known about the effects of extreme weather events on later stages of the breeding season and subsequent consequences on breeding performance. However, such effects are likely to affect breeding phenology only in subsequent seasons and then be diluted by other factors and may thus be difficult to see in our type of data. We therefore highlight that further research on the mechanisms through which climate change affects reproductive fitness in snowfinches and other cold-adapted species is urgently required^62^.

## Material and methods

### Study species

Snowfinches inhabit alpine and subnival habitats across the Palearctic and are resident to all major alpine mountain ranges of Central and Southern Europe with its nominotypical subspecies^63^. Snowfinches breed above the treeline from about 1.800 m asl, most densely at around 2450 m asl^46^ and primarily form loose colonies that nest in rock crevices from mid-May but secondarily also breed on anthropogenic structures (houses, ski lift poles) or in nest boxes^40,64^. Adults mostly provide their offspring with tipulid larvae collected along snow patch margins, later also including adult tipulids and other insects collected next to snow patches but are granivorous during the non-breeding period^40,65^.

### Bird data and data selection

We analysed a data set from the Swiss Alps spanning 20 years (Supplementary 3). The data set consisted of observations from an online database (https://www.ornitho.ch), population monitoring data (1999–2018)^46^, and own systematic brood monitoring data (2015–2018). Observers reported location, accuracy of location, date, number of individuals, and atlas codes (Supplementary 4) of a snowfinch sighting. Atlas codes indicate the birds’ behaviour during the breeding season, e.g. “adults with food for young” or “adults entering a nest site”^46^. Based on these atlas codes and known durations for nest building, incubation^40^, nestling period^64^ and fledgling period (pers. obs. & pers. comm Maria Delgado 2019), we calculated earliest, mean and latest possible hatching date per record (Supplementary 4), the distance between earliest and latest possible hatching date thus indicating hatching interval.

Observers might have differed in their habits to report the number of observed snowfinches. As multiple individuals per record either belonged to one or more broods, we discarded the reported number of individuals and thus treated each record as a single brood observation. To avoid bias caused by repeated observations of the same brood, we calculated the number of broods per 1 × 1 km grid cell and year. We first identified the minimum number of broods by finding mutually exclusive records of calculated hatching intervals. Per interval defined by these mutually exclusive records, we then chose a single interval from all overlapping intervals at random. If a single observer recorded more than one distinguishable brood on a single day, we included these unambiguously identified broods. For 1 × 1 km grid cells and years where we had systematic brood monitoring data available, we only included the latter data. This led to a total of 1135 recorded unique broods including 49 broods of precise hatching dates from the brood monitoring data and 1086 broods with hatching intervals from the citizen science data. Using the same method as for calculating hatching dates, we calculated nestling intervals and egg laying intervals.

### Spatiotemporal data

Per brood, we linked the information provided by the observers with the according biogeographic region, the elevation and calculated average observer day per kilometer square. We then used biogeographic region, elevation, year and average observer day for the spatiotemporal analysis. We used biogeographic regions defined by the Swiss Federal Office for the Environment^66^ to account for general climatic differences among regions within the Alps. Due to low sample sizes, Southern Tessin and Southern Alps were combined to region “Southern Alps” and Northern Alps and Prealps were combined to “Northern Alps” (Supplementary 5). Elevational data with a resolution of 200 × 200 m from the Swiss Federal Office for Topography (DHM25/200, swisstopo) was used. Because the nest location of broods was measured at different spatial accuracy, we computed both mean and standard deviation of elevation according to the reported precision of the nest location. We extracted precise elevation with a standard deviation of 0.5 m for all broods with known nests. For broods with a precision of up to 200 m, we used elevation per respective 200 × 200 m grid cell and calculated the standard deviation for the respective and all adjacent 200 × 200 m grid cells. The resulting 600 × 600 m cell corresponds to larger home range size of snowfinches rearing ^39^. For records of lower resolution, we calculated the mean of all 200 × 200 m grid cells in the respective 1 × 1 km grid cell and their standard deviation. We further calculated the average observer day per 1 × 1 km grid cell and year based on the number of persons who recorded bird observations from that grid cell per day between April 1st and August 31st.

### Environmental data

Temperature and precipitation data were provided by the Swiss Federal Institute for Forest, Snow and Lanscape Research WSL (Supplementary 6). We spatially integrated daily mean temperature and precipitation aggregated to 1 × 1 km grid cells based on a resolution of 100 × 100m^67^. We used a sliding window approach to identify the period with the highest Pearson’s product-moment correlation coefficient between hatching dates and temperature or precipitation, respectively. We investigated 7 windows of lengths between 1 and 90 days from January 1st to May 15th, corresponding to the 2.5 percentile of calculated mean hatching dates based on all records for which calculated hatching dates existed. Final windows for analysis were then chosen according to the following criteria: (1) the 20 highest correlations per window needed to fall on consecutive days and (2) the maximal correlation coefficient of the chosen window needed to exceed that of all other windows matching criteria (1). Accordingly, we chose mean February–March temperatures and mean April–May precipitation for later analysis. To account for the spatial uncertainty in the observational data, we calculated standard deviation of all aggregated 100 × 100 m grid cells per respective 1 × 1 km grid cell and used the average standard deviation per chosen window and record in our analysis. Gridded snow cover fraction was derived from daily observations at about 320 Swiss snow monitoring stations, assimilated into a distributed snowcover model following Magnusson et al.^68^. Within this model, sub-grid snow cover fraction was calculated according to Helbig et al.^69^ on a resolution of 1 × 1 km. We then used daily snow cover fraction per 1 × 1 km grid cell to to calculate snowmelt initiation (henceforth meltstart) and snowmelt completion (henceforth meltend). Meltstart was defined as the first day after maximal snow cover. Meltend was defined as the first day with minimal snow cover after snowmelt initiation. We defined winter intensity as the first principal component of annual mean winter temperatures of northern Switzerland above 1000 m asl between December and February (Swiss National Basic Climatological Network^70^) and mean total winter snow depth measured at Weissfluhjoch (46°49′59.797″N 9°48′23.183″E, 2691 m asl, Federal Office of Meteorology and Climatology, Supplementary 6).

### Statistical modelling

We first used linear mixed-effect models to model mean snowfinch hatching dates from all 1135 broods ranging from 1500 to 3077 m asl (Supplementary 7). We then modelled meltstart and meltend for all Swiss 1 × 1 km grid cells at the same elevational range and for all 1 × 1 km grid cells with snowfinch observations for a given year. All five models included elevation and year and additionally, a quadratic effect for elevation, an interaction term between elevation and year as fixed effects. In addition, we included year as a random effect to account for among-year variance that may be present additionally to the long-term trend. We used the function “lmer” in the lme4 package^71^ in the R statistical environment version 4.0.1^72^. We used the function “sim”^73^ to directly simulate 10.000 values from the joint posterior distribution of the model parameters while assuming p($\beta$) ∝ 1 as flat prior distributions for the coefficients and p($\sigma$) ∝ $\frac{1}{\sigma }$ for the variance parameters.

To obtain a description of the spatio-temporal patterns in hatching dates and to relate hatching dates to environmental variables we used two hierarchical models. In both models, we corrected for average observer day and accounted for the uncertainties in hatching dates. Further, in these models, we accounted for the variance in environmental variables that may be due to different precisions of the nest location by assuming a normal distribution with the mean and standard deviation measured of the environmental variables measured at the spatial resolution corresponding to the precision of the nest site location (see above).

In observational studies like ours, correlations among explanatory variables cannot be avoided. We therefore included all explanatory variables expected to affect responses in our models to determine partial correlations. Although including correlated predictors reduces power, removing one of two correlating effects would measure the effect of both predictors in a single correlation coefficient, but this estimate is not unbiased, as it is confounded by the other predictor. Because such partial effects do not correspond to the correlation in the data, we fitted two different models. One, using spatiotemporal parameters trying to describe patterns in the data, and another including spatiotemporal and environmental parameters, aiming to describe the mechanisms leading to the pattern. Nonetheless, Pearson’s correlation coefficients between predictors were below 0.6 except for the correlation between the linear and quadratic term for elevation (r = 0.998, Supplementary 8). Further, variance inflation factor tests on all predictors resulted in VIF-values being lower than 1.9, which are not problematic for the purpose of using models to describe the data, rather than to predict future hatching dates.

In the first model, we modelled hatching date as a function of biogeographic region, elevation, year and average observer day and included a quadratic term for elevation, an interaction term between elevation and year and a random year effect. We represented the hatching date per brood to be normally distributed with a mean equalling the mean hatching date and a standard deviation being a quarter of the hatching interval. Thus, we assume the true hatching date to fall within the hatching interval with a probability of 95%. Also, we accounted for uncertainty of nest site location by assuming a normal distribution for the elevation per brood with the mean and standard deviation of the elevation measured at the spatial resolution corresponding to the precision of the nest site location (see above).

In a second model, otherwise identical to the first, we further included winter intensity, mean spring temperature around the nest, precipitation and snowmelt timing to assess how environmental variables are related with snowfinch hatching date. As for elevation, we accounted for uncertainty in temperature and precipitation by assuming normal distributions with means and standard deviations as described above. For both models, we used Hamiltonian Monte Carlo simulation as implemented in Stan^74^ via the interface rstan^75^ to fit the models to the data^74^. We centred and scaled all numeric variables to ease convergence of the model fitting algorithm and for making some of the effect sizes comparable among each other. For both models, we simulated six Markov chains of length 10.000, each with the second half used to describe the posterior distributions of the model parameters. Priors were chosen identically for both models. For the intercept, we used a normal distribution with a mean corresponding to the overall mean hatching date (June 24th), and a standard deviation of 50 days. Of this prior distribution, 95% of the mass falls within the breeding season between May and August which we thus consider non-informative. For the standardised model coefficients, we used normal distributions with a mean of zero and a standard deviation of five days for both models and consider these weakly-informative priors. Year effects were assumed to follow a normal distribution with mean of zero and standard deviation of one. The prior distribution for the variance parameter ($\upsigma$) was described by a half-Cauchy distribution with a lower boundary at zero and a standard deviation of 20. The prior distribution for the among-year variance (${\upsigma }_{year}$) is described, by a half-Cauchy distribution with a location parameter set to zero and a scale parameter set to five. To check for convergence of the model fitting algorithm, we assessed the metrics and diagnostic plots provided by the r-packages rstan^75^ and shinystan^76^. For both models, Monte Carlo standard errors were less than 5%, R-hat values were below 1.01 and the number of effective samples was above 1500 for all parameters. To assess the goodness of fit of the models, we used visual posterior predictive checks which indicated the observed data for both models to be within the expected range of distributions. Spatial autocorrelation was examined based on the spatial distribution of the residuals and visualising the average semi-variance against distance classes^77,78^, but neither showed systematic patterns. To ensure the compatibility of the different data sets, we ran the analysis of both hierarchical models separately for the entire dataset and a dataset without the brood monitoring data. Due to the similarities of the citizen science data set and the monitoring data, we cannot think of any temporal patterns in the quality of data. All computations were conducted in a Bayesian framework using the statistical computing software R version 4.0.1^72^. We reported our results by presenting the mean of the posterior distribution as the point estimate and the 2.5% and 97.5% quantiles as a 95% compatibility interval (CI)^79^.

## Supplementary Information

Supplementary Information.

## Data Availability

The datasets generated and analysed during this study are available in the “vogelwarte.ch Open Repository And Archive” repository at 10.5281/zenodo.5464653.
