# Supplementary material for: Hatching phenology is lagging behind an advancing snowmelt pattern in a high-alpine bird
Source: Sci Rep. 2021 Nov 12;11:22191. doi: 10.1038/s41598-021-01497-8 (PMC8589975; doi:10.1038/s41598-021-01497-8)
Supplement: Supplementary file 1 — Supplementary Information. [file 41598_2021_1497_MOESM1_ESM.pdf]

# Hatching phenology is lagging behind an advancing snowmelt pattern in a high-alpine bird

**Christian Schano[1,2,*], Carole Niffenegger[1], Tobias Jonas[3], Fränzi Korner-Nievergelt[1]**

[1] Swiss Ornithological Institute, Sempach, 6204, Switzerland

[2] University of Zurich, Department of Evolutionary Biology and Environmental Studies, 8057, Zurich, Switzerland

[3] WSL Institute for Snow and Avalanche Research SLF, Snow Hydrology, 7260, Davos Dorf, Switzerland

* christian.schano@vogelwarte.ch

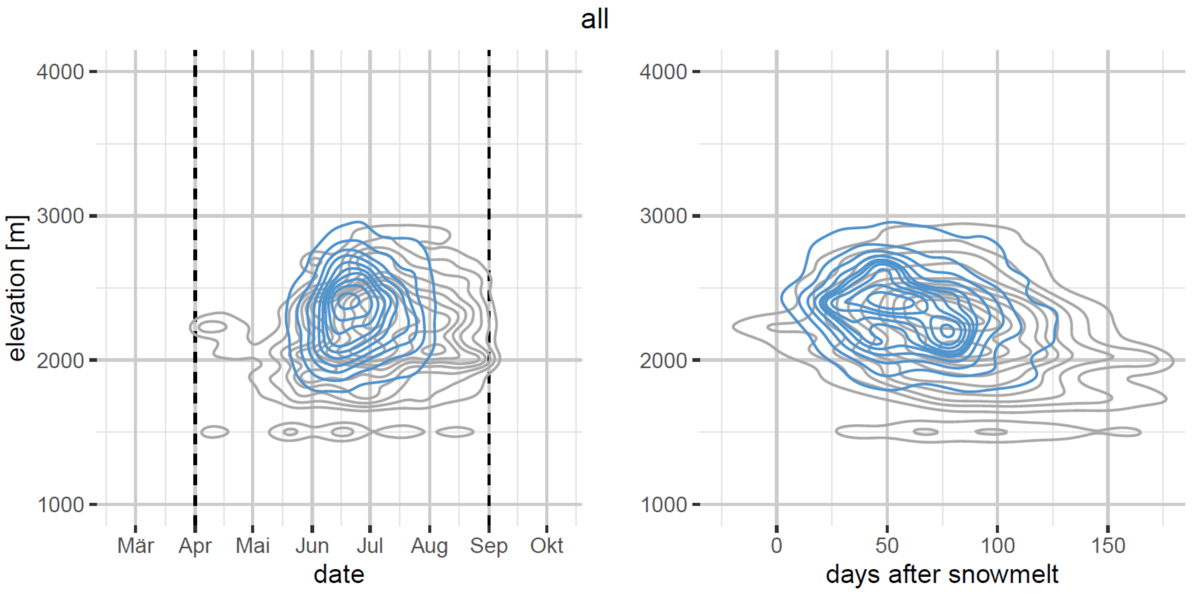


Supplementary 1 Kernel densities with 10% mass distribution increments ranging from 95% to 5% for snowfinch hatching dates (blue) and observer presence (grey) by date (left) and relative to snowmelt (right) and elevation.

Supplementary 2 Residuals of the environmental model plotted against the year showing mean (horizontal, fat black lines), interquartile range (boxes), 1.5 times interquartile range (whiskers) and outliers (points) per year.

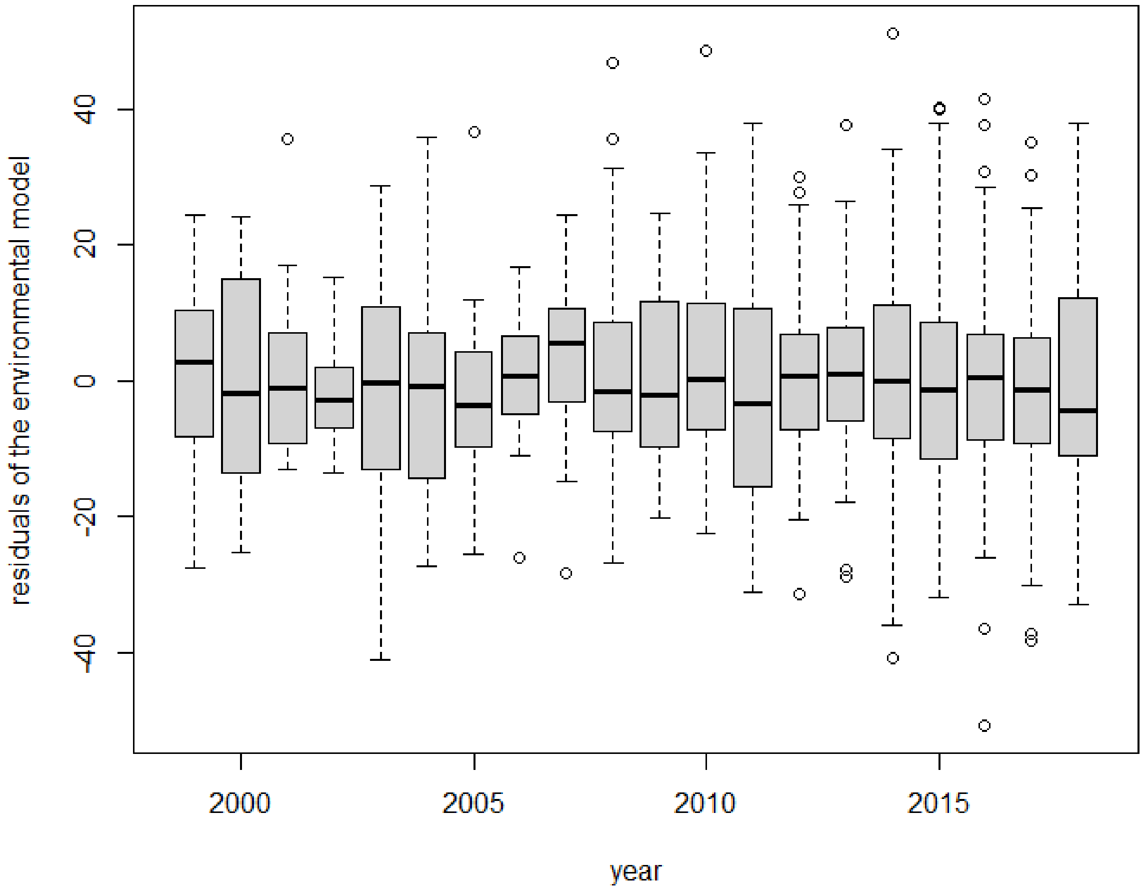

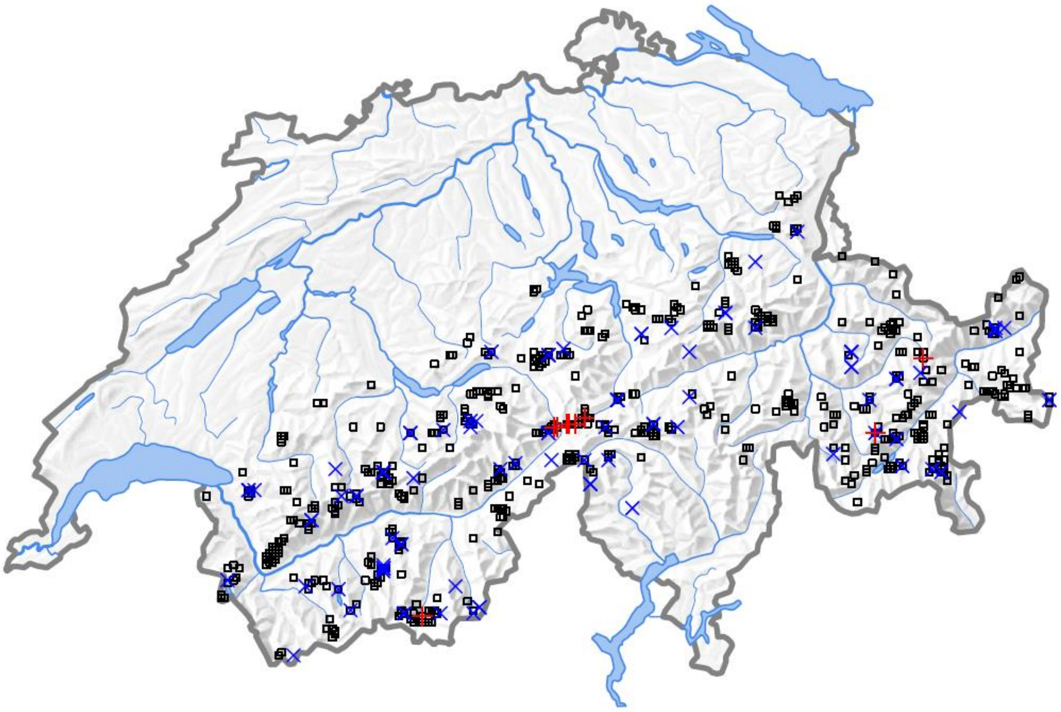

Supplementary 3 Map of Switzerland with observations of snowfinches 1999 - 2018. 1x1km grid cells with snowfinch observations from citizen scientists (black squares), standardized monitoring programs (blue ”xx”) and the brood monitoring program data (red “++”) where calculating hatching dates was possible (© swisstopo).

Supplementary 4 Atlas codes, describtions and formulas used to calculate hatching dates. Abbreviations stand for (F) fledgling period – 7 days, (H) hatching period – 21 days, (I) incubation period – 13 days, and (N) nest building period – 13 days. Mean hatching dates per observation are based on the arithmetic mean between earliest calculated (hatchmin) and latest calculated (hatchmax) hatching date.

| Atlas code | Description | Associated life event | Formula |
|---|---|---|---|
| 8 | Alarming adult indicating a nest or chicks close by | I or H | hatchmin = doyobs – H + F – 1<br>hatchmax = doyobs + I |
| 10 | Adult with nesting material or building nest | N | hatchmin = doyobs + I + 1<br>hatchmax = doyobs + N + I |
| 11 | Adult with distraction-display or injury-feigning | H | hatchmin = doyobs – H + F – 1<br>hatchmax = doyobs |
| 12 | Used nest from current season | I or H | hatchmin = doyobs – H + F – 1<br>hatchmax = doyobs + I |
| 13 | Recently fledged young | H or F | hatchmin = doyobs – H + F – 1<br>hatchmax = doyobs - H |
| 14 | Adults entering or leaving a potentially occupied nest | I or H | hatchmin = doyobs – H – 1<br>hatchmax = doyobs + I |
| 15 | Adult carrying fecal sac of chick | H | hatchmin = doyobs – H - 1<br>hatchmax = doyobs |
| 16 | Adult carrying food for chicks | H | hatchmin = doyobs – H - 1<br>hatchmax = doyobs |
| 18 | Nest with breeding adult | I | hatchmin = doyobs + 1<br>hatchmax = doyobs + I |
| 19 | Nest with eggs or chicks | I or H | hatchmin = doyobs – H – 1<br>hatchmax = doyobs + I |

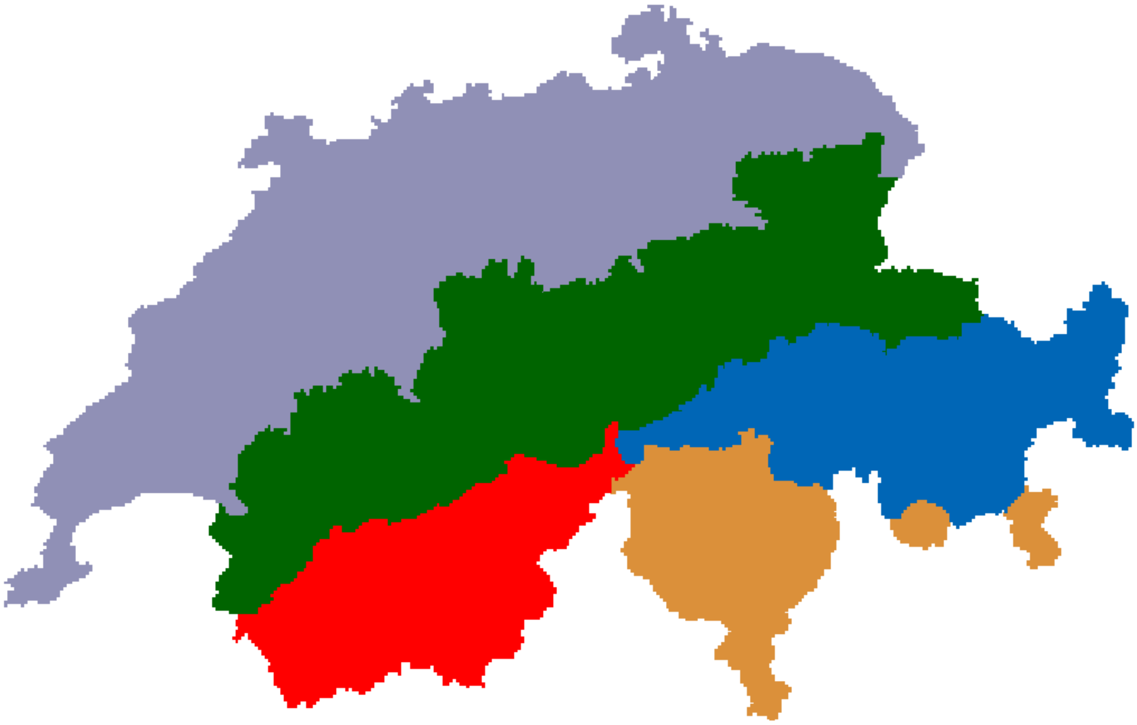

Supplementary 5 Biogeographic regions used for analysis of spatiotemporal patterns in snowfinch hatching dates (© swisstopo). Eastern Alps (blue), Northern Alps (Green), Southern Alps (orange), Western Alps (red) and excluded regions (grey).

Supplementary 6 Yearly mean temperature (a), precipitation (b) and winter intensity (c) for all kilometer squares with snowfinch observations between 1999 and 2018 indicating means (circles), ± 1.96 standard deviations (grey vertical lines), connected with a dotted line. We did not plot standard deviations for winter harshness, since we only had single estimates for the entire study area

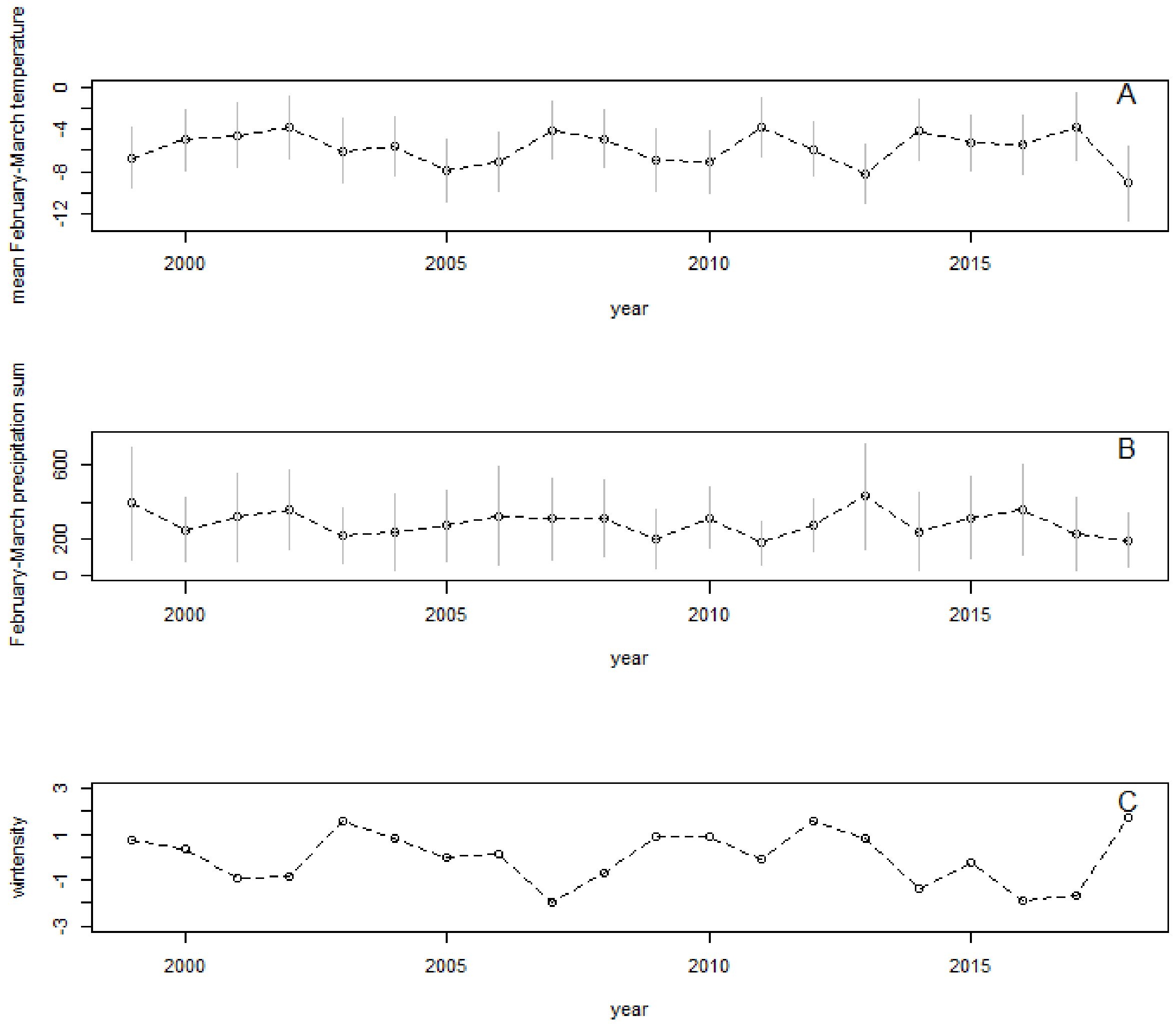

Supplementary 7 Model description, used data and formulas for models used in the analysis. Model coefficients (β), elevation (elev), mean observer day (obsday), April-May precipitation sum (prcp), February-March average daily temperature (tave), winter intensity (winter), year (year, yeareff), among-year variance ($\sigma_{year}$)

| Model description | Used data | Formula |
|---|---|---|
| Long-term trends in hatching date | Hatching date for 1x1km grid cells with snowfinch observations in current season. | $\sim \beta_1 * \mathrm{elev}_i + \beta_2 * \mathrm{elev^2}_i + \beta_3 * \mathrm{year}_i + \beta_4 * \mathrm{elev}_i * \mathrm{year}_i + \sigma_{year} * \mathrm{yeareff}_{year[i]}$ |
| Long-term trends in snowmelt start | Snowmelt start for 1x1km grid cells with snowfinch observations in current season and calculated for all 1x1km grid cells in Switzerland above 1500 m asl. | $\sim \beta_1 * \mathrm{elev}_i + \beta_2 * \mathrm{elev^2}_i + \beta_3 * \mathrm{year}_i + \beta_4 * \mathrm{elev}_i * \mathrm{year}_i + \sigma_{year} * \mathrm{yeareff}_{year[i]}$ |
| Long-term trends in snowmelt end | Snowmelt end for 1x1km grid cells with snowfinch observations in current season and calculated for all 1x1km grid cells in Switzerland above 1500 m asl. | $\sim \beta_1 * \mathrm{elev}_i + \beta_2 * \mathrm{elev^2}_i + \beta_3 * \mathrm{year}_i + \beta_4 * \mathrm{elev}_i * \mathrm{year}_i + \sigma_{year} * \mathrm{yeareff}_{year[i]}$ |
| Spatiotemporal patterns in hatching date | Snowfinch data from 1135 breeding records | $\sim \beta_{region[i]} + \beta_1 * \mathrm{elev}_i + \beta_2 * \mathrm{obsday}_i + \beta_3 * \mathrm{year}_i + \beta_4 * \mathrm{elev^2}_i + \beta_5 * \mathrm{year}_i * \mathrm{elev}_i + \sigma_{year} * \mathrm{yeareff}_{year[i]}$ |

| | | |
|---|---|---|
| Spatiotemporal and environmental variables affecting hatching date | Snowfinch data from 1135 breeding records | $\sim \beta_{region[i]} + \beta_1 * elev_i + \beta_2 * obsday_i + \beta_3 * year_i + \beta_4 * elev^2{}_i + \beta_5 * year_i * elev_i + \beta_6 * winter_i + \beta_7 * prcp_i + \beta_8 * melt_i + \beta_9 * tave_i + \sigma_{year} * yeareff_{year[i]}$ |

Supplementary 8 Pearson`s correlation coefficients between all predictors used for the spatiotemporal and the environmental model.

| | elevation | observer day | year | winter intensity | snowmelt | precipitation | temperature | elevation$^2$ |
|---|---|---|---|---|---|---|---|---|
| elevation | - | 0.224 | 0.217 | -0.161 | 0.347 | -0.263 | -0.573 | 0.998 |
| observer day | | - | 0.429 | 0.117 | 0.124 | 0.046 | -0.260 | 0.226 |
| year | | | - | -0.288 | 0.031 | -0.059 | -0.081 | 0.211 |
| Winter intensity | | | | - | -0.033 | -0.056 | -0.557 | 0.002 |
| snowmelt | | | | | - | 0.266 | -0.358 | 0.337 |
| precipitation | | | | | | - | 0.039 | -0.267 |
| temperature | | | | | | | - | -0.570 |
| elevation$^2$ | | | | | | | | - |
